# Supplementary material for: Characterization of Class-3 Semaphorin Receptors, Neuropilins and Plexins, as Therapeutic Targets in a Pan-Cancer Study
Source: Cancers (Basel). 2020 Jul 6;12(7):1816. doi: 10.3390/cancers12071816 (PMC7409005; doi:10.3390/cancers12071816)
Supplement: Supplementary file 1 [file cancers-12-01816-s001.pdf]

cancers

MDPI

Article

Characterization of Class-3 Semaphorin Receptors, Neuropilins and Plexins, as Therapeutic Targets in a Pan-Cancer Study

Xiaoli Zhang, Shuai Shao [2] and Lang Li

Supplementary Materials:

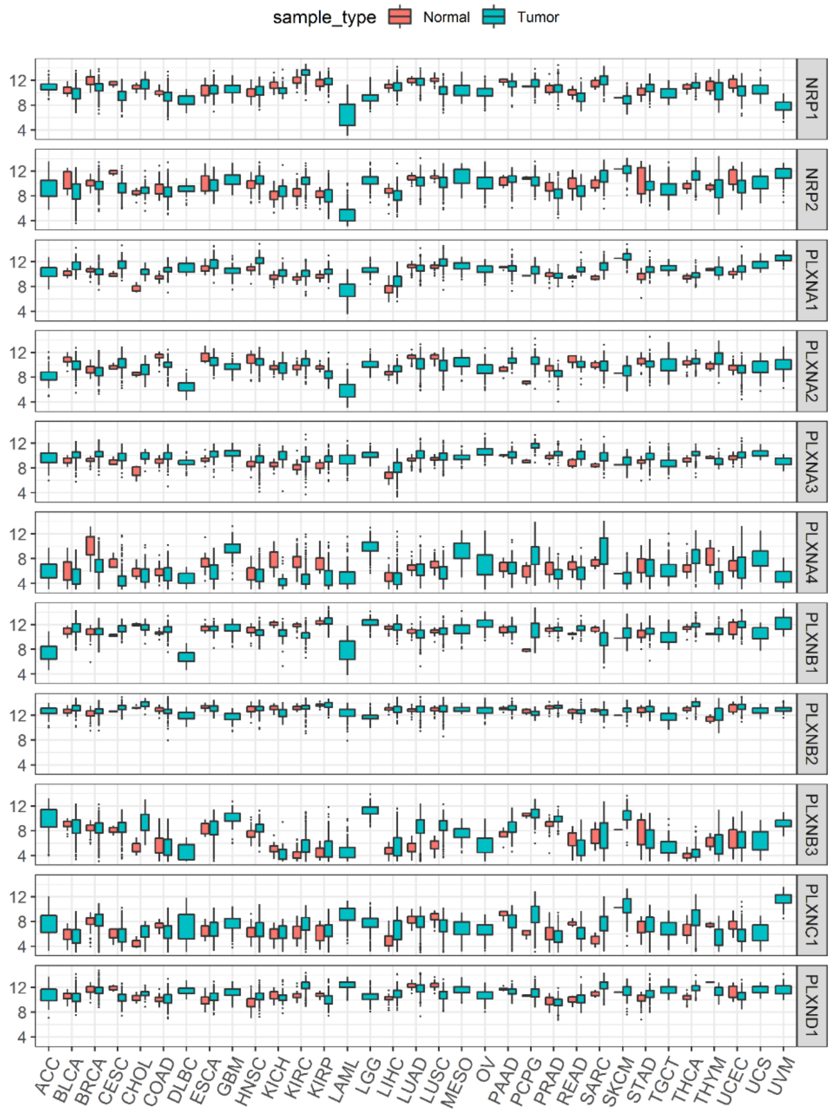

Figure S1. Boxplots to show the expression levels of NRPs and PLXNs in cancerous and adjacent normal tissues for all 33 cancer types. Boxplots represent the distribution of the NRP and PLXN gene expression levels (log2[RSEM normalized values relative to TBP]) in primary tumour and normal tissues (if available) of different cancer types for each of the genes. The band inside the box is the median expression values for the gene. Comparisons between normal and tumour expression values were performed with linear mixed effects models. A p-value<0.005 after controlling 1 false positive among all the tests was considered as significance (53).

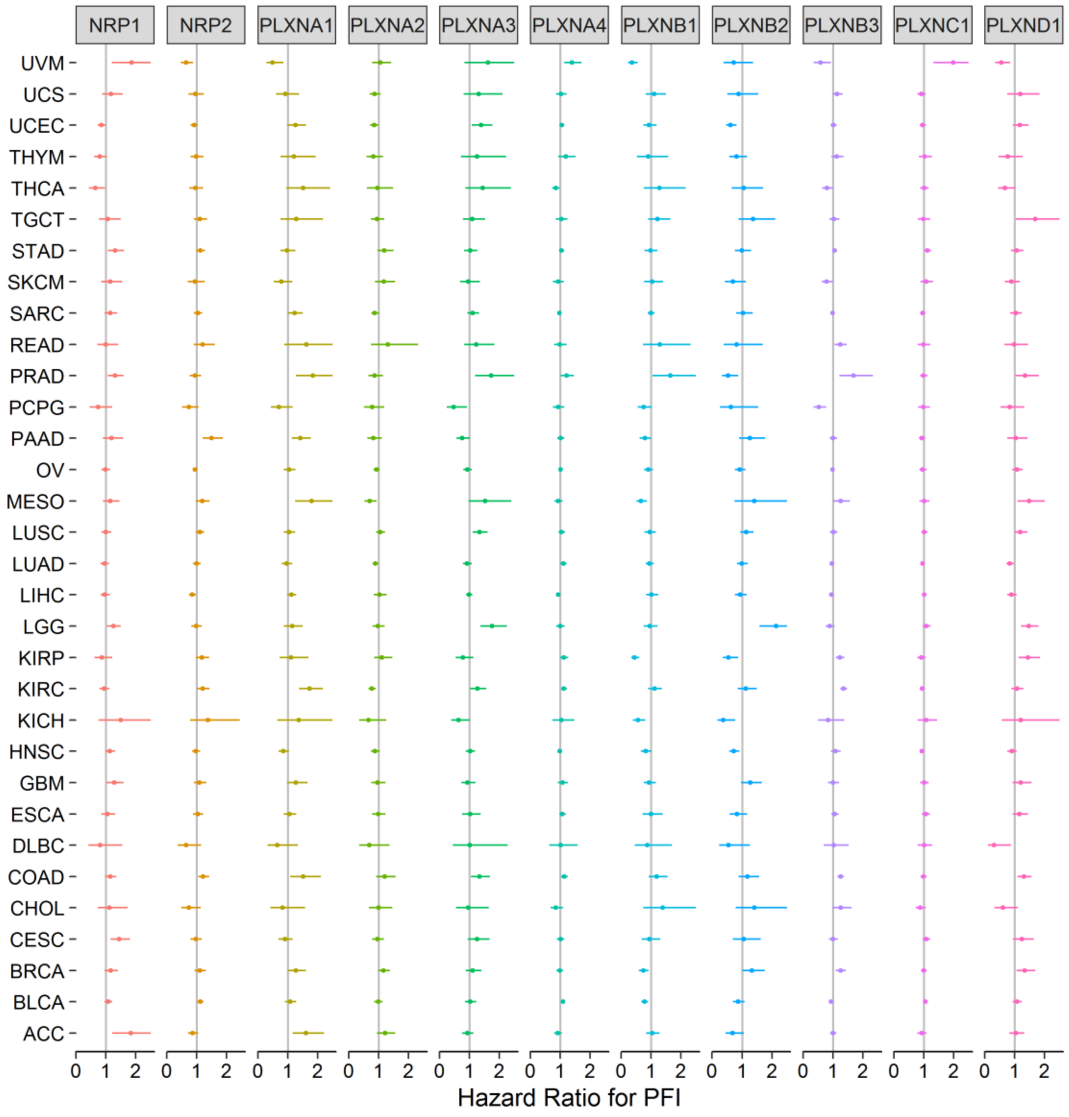

Figure S2. Association of the expressions of NRPs and PLXNs with patient progression free interval (PFI) for different cancer types. The forest plots with the hazard ratios and 95% confidence intervals for PFI for different cancer types to show survival advantage and disadvantage with increased gene expression of NRPs and PLXNs. Univariate Cox proportional hazard regression models were used for the association tests. P<0.05 was considered as significance.

A

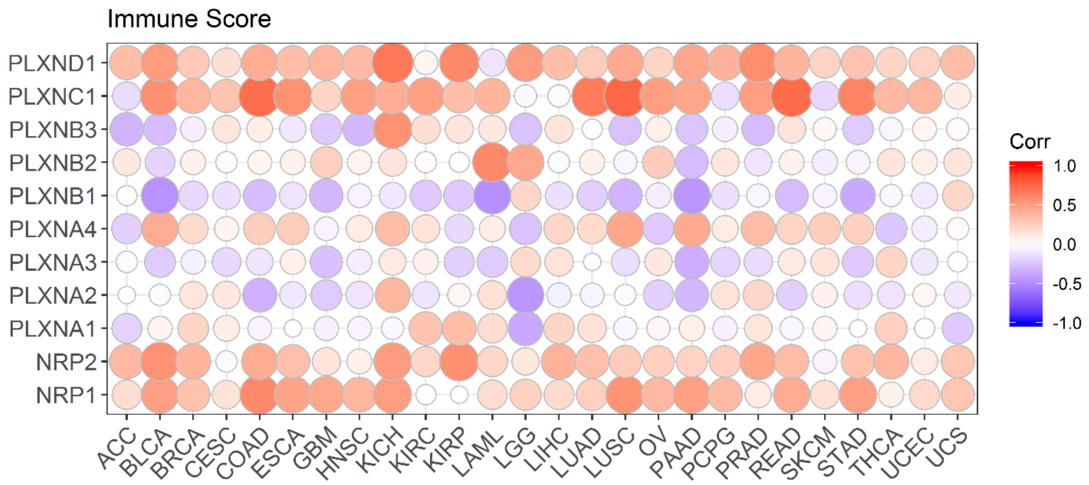

B

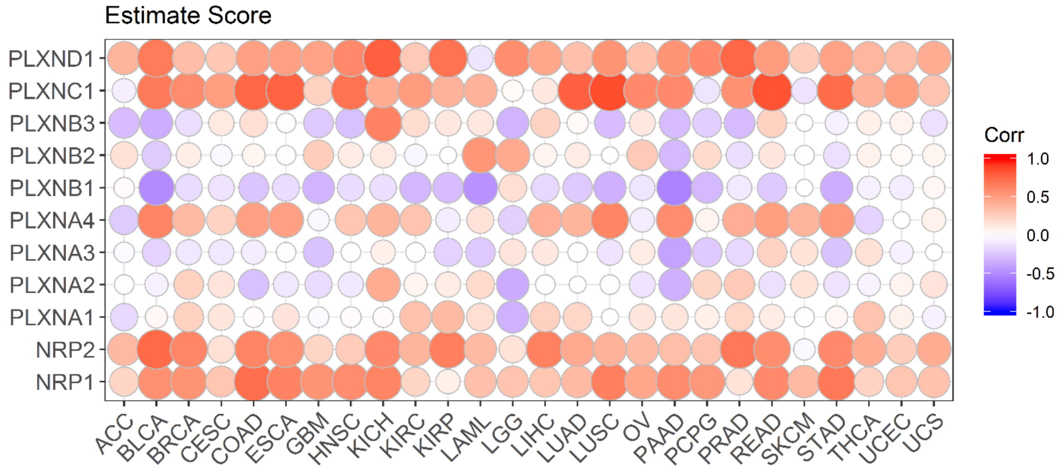

Figure S3. Correlation matrix plots to show the association between the expressions of NRPs and PLXNs and immune score and estimate scores of 25 different cancer types based on ESTIMATE algorithm. (A). Immune score, and (B). Estimate score. Spearman correlation was used for testing. The size of the dots stands for the absolute value of the correlation coefficients. The bigger the size is, the higher the correlation is (higher absolute correlation coefficient). P<1/ (11 genes x 26 cancer types) =0.0034 as significance.

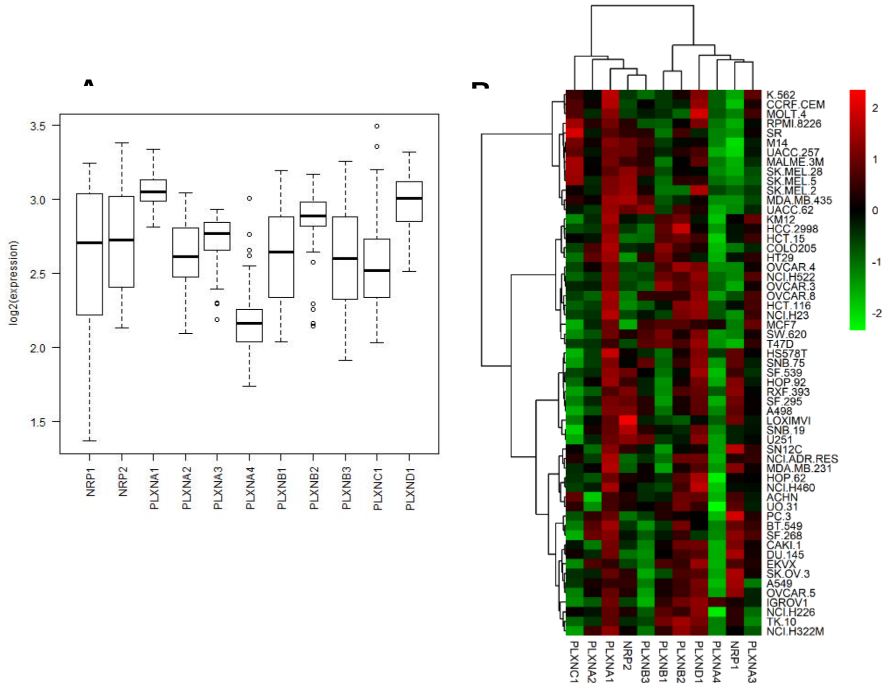

Figure S4. Expression of NRPs and PLXNs in NCI-60 cell lines. (A). Boxplots to show the distribution of the expression of NRPs and PLXNs across NCI-60 cell lines. (B). Heatmap to show the expression of NRPs and PLXNs within each individual cell lines using NCI-60 cell line data.

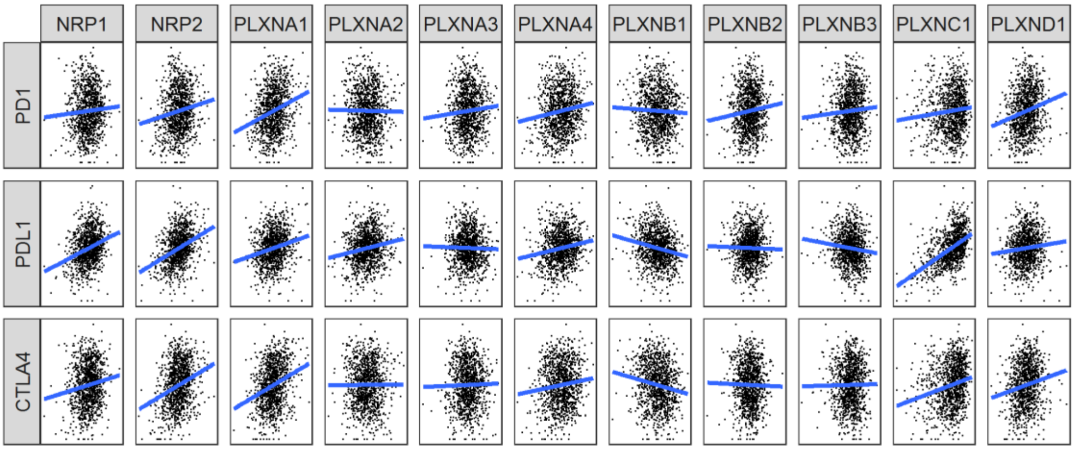

Figure S5. Scatter plots to show the correlation of the expression of NRPs and PLXNs with the level of immune checkpoint molecules PD1/PDL1 and CTLA-4 in breast cancer tumors. P< 1/ (11 genes x 3 immune blockade molecules) = 0.03 as significance.

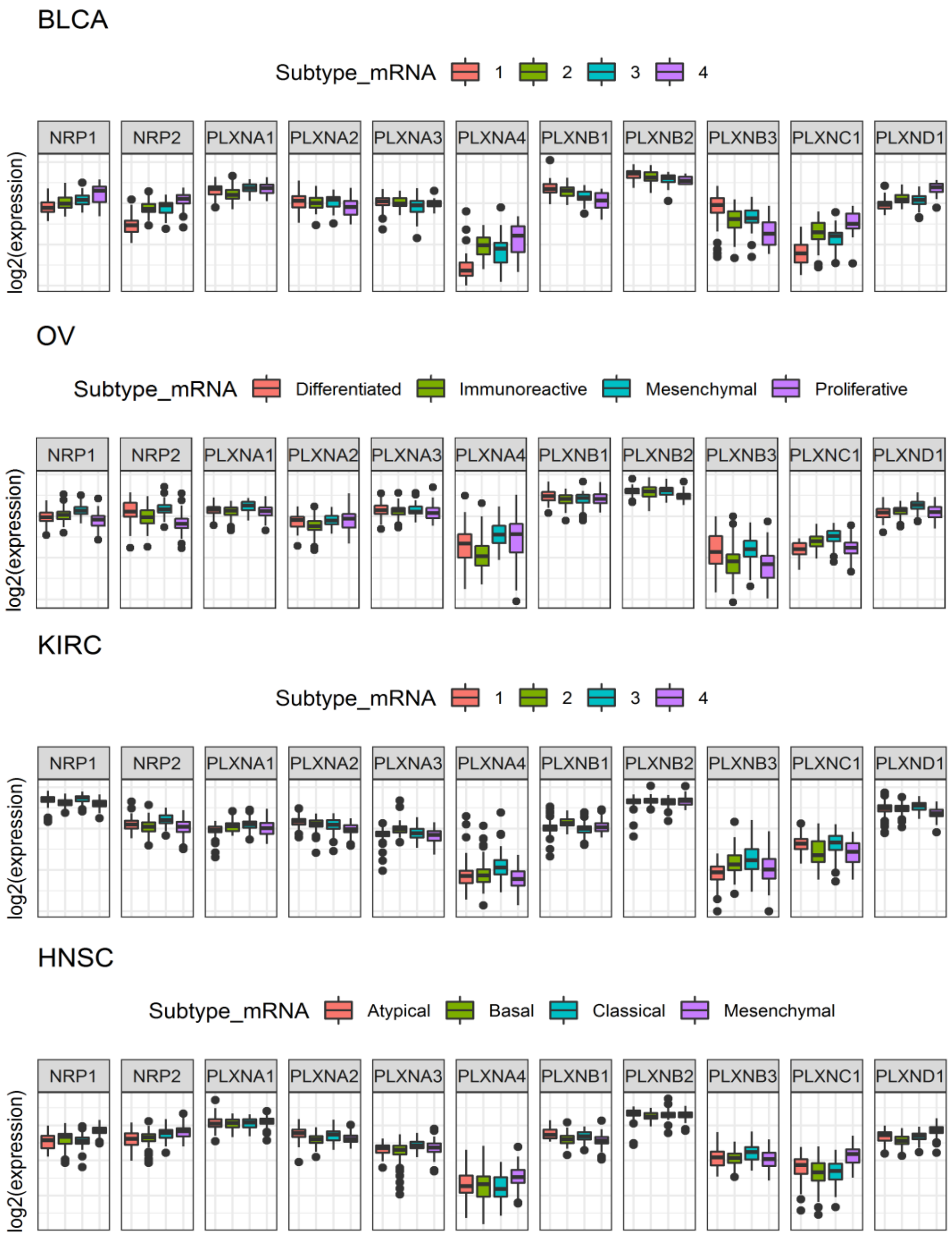

Figure S6. Boxplots to show the gene expression of NRPs and PLXNs in different molecular subtypes of BLCA, OV, KIRC, and HNSC tested with ANOVA (P<.0001

cancers

MDPI

Table S1. Summary of TCGA pan-cancer data. This table includes the number of total samples, number of blood and primary tumors, metastatic tumors (met tumor), and adjacent normal tissues (normal), as well as number of overall survival (OS) or progression free interval (PFI) events ("1") or censored ("0") patients .

Primary Disease Type	Tcgaid	Tissue Types					Os			Pfi		
		Normal	Blood Tumor	Primary Tumor	Met Tumor	Total	0	1	Total	0	1	Total
**adrenocortical cancer**	**ACC**	0	0	79	0	79	51	28	79	38	41	79
bladder urothelial carcinoma	**BLCA**	19	0	408	0	427	229	179	408	234	174	408
breast invasive carcinoma	**BRCA**	113	0	1095	7	1215	944	151	1095	950	145	1095
cervical & endocervical cancer	**CESC**	3	0	304	2	309	233	71	304	233	71	304
cholangiocarcinoma	**CHOL**	9	0	36	0	45	18	18	36	16	20	36
colon adenocarcinoma	**COAD**	41	0	449	1	491	348	101	449	329	120	449
diffuse large B-cell lymphoma	**DLBC**	0	0	48	0	48	39	9	48	36	12	48
esophageal carcinoma	**ESCA**	11	0	184	1	196	107	77	184	97	87	184
glioblastoma multiforme	**GBM**	0	0	153	0	153	32	121	153	33	120	153

head & neck squamous cell carcinoma	**HNSC**	44	0	520	2	566	300	220	520	323	197	520
kidney chromophobe	**KICH**	25	0	66	0	91	56	10	66	54	12	66
kidney clear cell carcinoma	**KIRC**	72	0	534	0	606	359	175	534	372	162	534
kidney papillary cell carcinoma	**KIRP**	32	0	291	0	323	247	44	291	232	59	291
acute myeloid leukemia	**LAML**	0	173	0	0	173	59	114	173	0	0	0
brain lower grade glioma	**LGG**	0	0	515	0	515	390	125	515	323	192	515
liver hepatocellular carcinoma	**LIHC**	50	0	371	0	421	241	130	371	191	180	371
lung adenocarcinoma	**LUAD**	59	0	515	0	574	328	187	515	306	209	515
lung squamous cell carcinoma	**LUSC**	51	0	501	0	552	285	216	501	354	147	501
mesothelioma	**MESO**	0	0	87	0	87	13	74	87	26	61	87
ovarian serous cystadenocarcinoma	**OV**	0	0	304	0	304	121	183	304	94	210	304
pancreatic adenocarcinoma	**PAAD**	4	0	178	1	183	85	93	178	74	104	178

pheochromocytoma & paraganglioma	**PCPG**	3	0	182	2	187	175	7	182	160	22	182
prostate adenocarcinoma	**PRAD**	52	0	497	1	550	487	10	497	404	93	497
rectum adenocarcinoma	**READ**	10	0	159	0	169	136	23	159	125	34	159
sarcoma	**SARC**	2	0	259	1	262	161	98	259	121	138	259
skin cutaneous melanoma	**SKCM**	1	0	103	369	473	74	29	103	66	37	103
stomach adenocarcinoma	**STAD**	35	0	415	0	450	255	160	415	280	135	415
testicular germ cell tumor	**TGCT**	0	0	139	0	139	135	4	139	99	40	139
thyroid carcinoma	**THCA**	59	0	505	8	572	489	16	505	453	52	505
thymoma	**THYM**	2	0	120	0	122	111	9	120	99	21	120
uterine corpus endometrioid carcinoma	**UCEC**	22	0	532	0	554	445	87	532	412	120	532
uterine carcinosarcoma	**UCS**	0	0	57	0	57	22	35	57	20	37	57
uveal melanoma	**UVM**	0	0	80	0	80	57	23	80	50	30	80
	**Total**	719	173	9686	395	10973	7032	2827	9859	6604	3082	9686

Table S2. Correlation between mRNA and protein level expression of SEMA3s and their receptors in breast, ovarian, and colorectal cancers. The cells left blank indicate that the genes were not detected by proteomics study; therefore, no correlation between mRNA and protein was calculated. P< 1/ (18 genes x 3 cancer types) = 0.018 as significant.

**Correlation between mRNA and protein level expression of SEMA3s and their receptors**						
	Breast Cancer (BRCA)		Ovarian Cancer (OV)		Colon Cancer (COAD)	
**Gene_ID**	**Correlation coefficient (r)**	*p*-values	Correlation coefficient (r)	*p*-values	Correlation coefficient (r)	*p*-values
NRP1	0.5427	2.00E-07	0.62984	<.0001	0.23852	0.074
NRP2	0.5794	1.80E-08	0.52814	<.0001		
PLXNA1	0.2948	7.94E-03	0.15252	0.1222	0.05684	0.6475
PLXNA2	0.0524	0.65738	0.17209	0.0792		
PLXNA3	0.2789	1.54E-02	0.2393	0.0209		
PLXNA4	0.1352	0.29488	0.1211	0.2325	0.13488	0.3172
PLXNB1	0.1928	0.10972	0.48416	<.0001	-0.00235	0.9862
PLXNB2	0.5042	1.90E-06	0.48187	<.0001	-0.06725	0.6191
PLXNB3	0.4031	4.57E-02				
PLXNC1	0.3868	3.90E-04	0.45311	<.0001		
PLXND1	0.3288	2.91E-03	0.20183	0.039	0.17207	0.2006
SEMA3A	-0.0043	0.97355				
SEMA3B	0.558	2.00E-07	0.21162	0.2086		
SEMA3C	0.6044	2.90E-09	0.5563	0.0002		
SEMA3D	-0.1233	0.29205				
SEMA3E						
SEMA3F	0.613	9.30E-08	0.38636	0.0264		
SEMA3G					0.17207	0.2006

Table S3. Correlation between SEMA3 receptors NRPs and PLXNs and SEMA3 key signal transducers averaged across all 33 cancer types tested by Pearson correlation. For each correlation, the upper cell is the correlation coefficient (r) and the lower cell is the P-value for the correlation test. $p < 1/(11 \text{ genes} \times 14 \text{ key transducers}) = 0.0064$ as significance.

**Pearson Correlation Coefficients, N = 10254**											
	**NRP1**	**NRP2**	**PLXNA1**	**PLXNA2**	**PLXNA3**	**PLXNA4**	**PLXNB1**	**PLXNB2**	**PLXNB3**	**PLXNC1**	**PLXND1**
**ERBB2**	0.190	0.028	0.088	0.125	0.189	-0.095	0.349	0.413	-0.082	-0.196	-0.109
	<.0001	0.005	<.0001	<.0001	<.0001	<.0001	<.0001	<.0001	<.0001	<.0001	<.0001

**KDR**	0.673	0.461	0.002	0.378	0.054	0.266	-0.036	0.108	0.003	0.286	0.395
	<.0001	<.0001	0.858	<.0001	<.0001	<.0001	0.000	<.0001	0.758	<.0001	<.0001
**PTK7**	0.041	0.238	0.375	0.133	0.293	0.155	0.138	0.160	0.142	0.007	0.060
	<.0001	<.0001	<.0001	<.0001	<.0001	<.0001	<.0001	<.0001	<.0001	0.450	<.0001
**MET**	0.364	0.241	0.218	0.317	-0.113	-0.231	0.135	0.394	-0.273	-0.006	0.012
	<.0001	<.0001	<.0001	<.0001	<.0001	<.0001	<.0001	<.0001	<.0001	0.560	0.223
**MST1R**	-0.016	0.019	0.246	0.228	0.157	-0.109	0.155	0.269	-0.086	-0.138	-0.075
	0.102	0.061	<.0001	<.0001	<.0001	<.0001	<.0001	<.0001	<.0001	<.0001	<.0001
**PTK2B**	-0.120	-0.068	-0.070	0.088	0.100	0.106	-0.038	0.114	-0.023	0.111	0.065
	<.0001	<.0001	<.0001	<.0001	<.0001	<.0001	0.000	<.0001	0.021	<.0001	<.0001
**SRC**	0.007	0.077	0.460	0.248	0.218	-0.100	0.212	0.257	0.219	-0.137	-0.207
	0.477	<.0001	<.0001	<.0001	<.0001	<.0001	<.0001	<.0001	<.0001	<.0001	<.0001
**FYN**	0.112	0.458	0.087	0.184	0.042	0.489	-0.005	-0.217	0.095	0.409	0.423
	<.0001	<.0001	<.0001	<.0001	<.0001	<.0001	0.602	<.0001	<.0001	<.0001	<.0001
**FES**	0.041	0.050	-0.064	-0.080	-0.072	0.032	-0.169	0.106	-0.115	0.183	0.472
	<.0001	<.0001	<.0001	<.0001	<.0001	0.001	<.0001	<.0001	<.0001	<.0001	<.0001
**PTK2**	0.312	0.371	0.333	0.317	0.120	0.122	0.216	0.022	0.217	0.044	-0.092
	<.0001	<.0001	<.0001	<.0001	<.0001	<.0001	<.0001	0.026	<.0001	<.0001	<.0001
**RND1**	0.156	-0.040	-0.232	0.088	0.010	0.129	0.212	-0.038	0.083	-0.057	-0.007
	<.0001	<.0001	<.0001	<.0001	0.307	<.0001	<.0001	0.000	<.0001	<.0001	0.456
**RRAS**	0.352	0.239	0.088	0.190	0.088	-0.011	0.059	0.359	-0.115	-0.152	0.118
	<.0001	<.0001	<.0001	<.0001	<.0001	0.281	<.0001	<.0001	<.0001	<.0001	<.0001
**RAC1**	0.056	0.143	0.254	0.167	-0.023	-0.059	0.097	0.010	0.114	-0.197	-0.181
	<.0001	<.0001	<.0001	<.0001	0.019	<.0001	<.0001	0.290	<.0001	<.0001	<.0001
**RHOA**	0.094	0.158	-0.041	-0.069	-0.045	0.090	-0.103	-0.092	-0.187	0.195	0.198
	<.0001	<.0001	<.0001	<.0001	<.0001	<.0001	<.0001	<.0001	<.0001	<.0001	<.0001

Table S4. Correlation between SEMA3 receptors and immune checkpoint molecules across all 33 cancer types tested with Pearson Correlation test. Note: r=correlation coefficient, and p=p-values for the correlation test. P<1/(11 genes x 3 immune checkpoint molecules)= 0.03 as significance.

Correlation between SEMA3 receptors and immune checkpoint molecules across all cancer types						
**SEMA receptors**	**PD1_r**	**PD1_corr_p**	**PDL1_r**	**PDL1_corr_p**	**CTLA4_r**	CTLA4_corr_p
NRP1	0.17647	<.0001	0.2829	<.0001	0.11932	<.0001
NRP2	0.08991	<.0001	0.33764	<.0001	0.11504	<.0001
PLXNA1	0.16886	<.0001	0.27114	<.0001	0.2624	<.0001
PLXNA2	0.03641	0.0003	0.25319	<.0001	0.0484	<.0001
PLXNA3	-0.09337	<.0001	-0.02181	0.0302	-0.06152	<.0001
PLXNA4	-0.10993	<.0001	0.02474	0.014	-0.10632	<.0001
PLXNB1	-0.21103	<.0001	-0.19803	<.0001	-0.24992	<.0001
PLXNB2	0.14452	<.0001	0.07199	<.0001	0.079	<.0001
PLXNB3	-0.17147	<.0001	-0.02223	0.0272	-0.06854	<.0001
PLXNC1	0.09588	<.0001	0.3316	<.0001	0.14099	<.0001
PLXND1	0.24898	<.0001	0.15345	<.0001	0.14215	<.0001

Table S5. The list of significantly correlated genes and drugs with |r|>0.4 and p-values<0.005.

GeneID	DrugID	DrugName	Drug targets/ mechanism of action	Correlation coefficient	P-values
NRP1	638850	7-Hydroxystaurosporine	Phosphokinases inhibitor	0.528706839	4.71E-05
NRP1	773263	Bafetinib	Dual Bcr-Abl and Src family kinases Lck and Lyn inhibitor	-0.493224168	0.000112
NRP1	26271	Cyclophosphamide	Target on DNA	-0.466085161	0.001096
NRP1	732517	Dasatinib	dual BCR/ABL and Src family tyrosine kinase inhibitor	0.549766043	9.45E-06
NRP1	759877	Dasatinib	dual BCR/ABL and Src family tyrosine kinase inhibitor	0.599804438	8.19E-07
NRP1	755892	Hydrastinine HCl	can be used as a haemostatic agent[	-0.419444511	0.001163
NRP1	354462	Hypothemycin	unknown	-0.526380669	2.60E-05
NRP1	656576	Midostaurin	PKCalpha, VEGFR2, KIT, PDGFR and WT and/or mutant FLT3 tyrosine kinases	0.479607427	0.00016

NRP1	747599	Nilotinib	tyrosine kinase activity of the BCR-ABL protein	-0.450694753	0.000491
NRP1	758706	Simvastatin	a lipid-lowering drug	0.416837238	0.001257
NRP1	633782	Simvastatin	a lipid-lowering drug	0.511686779	0.00087
NRP1	618487	Staurosporine	Tyrosine protein kinase	0.631289621	1.40E-07
NRP1	701852	Vorinostat	histone deacetylases HDAC1, HDAC2 and HDAC3 (Class I) and HDAC6 (Class II)	-0.432041795	0.000791
NRP2	354258	8-Chloro-adenosine	Induce AMP-activated protein kinase (AMPK) pathway leading to autophagy	-0.465792463	0.000261
NRP2	766270	ABT-199	BCL-2 inhibitor	0.456814099	0.000455
NRP2	764134	Dabrafenib	BRAF mutant inhibitor	0.456657271	0.000973
NRP2	677083	okadaic acid	neurotoxin and phosphatase inhibitor	0.419808878	0.00115
NRP2	627168	Pyrazoloacridine	acridine anticancer agent	-0.419571926	0.002992
NRP2	741078	Selumetinib	KRAS/MEK inhibitor	0.409108803	0.001579
NRP2	761431	Vemurafenib	Mutated BRAF inhibitor	0.443343038	0.000553
PLXNA1	354258	8-Chloro-adenosine	Induce AMP-activated protein kinase (AMPK) pathway leading to autophagy	-0.456003819	0.000364
PLXNA1	773263	Bafetinib	Dual Bcr-Abl and Src family kinases Lck and Lyn inhibitor	0.426096329	0.001059
PLXNA1	756738	Digoxin	inhibition of the sodium potassium adenosine triphosphatase (Na+/K+ ATPase)	-0.455916486	0.000531
PLXNA1	354462	Hypothemycin	inhibitor of MEK	0.406659755	0.001695
PLXNA1	757087	Nitrogen mustard	cytotoxic organic compounds with the chloroethylamine functional group	-0.439265975	0.000707
PLXNA1	677083	okadaic acid	neurotoxin and phosphatase inhibitor	0.44763607	0.000481
PLXNA1	741078	Selumetinib	KRAS/MEK inhibitor	0.406631924	0.001696
PLXNA1	761431	Vemurafenib	BRAF mutant inhibitor	0.521642937	3.17E-05
PLXNA2	354258	8-Chloro-adenosine	Induce AMP-activated protein kinase (AMPK) pathway leading to autophagy	-0.509922443	5.08E-05
PLXNA2	1390	Allopurinol	Inhibitor of xanthine oxidase	-0.404654448	0.001979
PLXNA2	758186	Azacitidine	inhibits RNA methylation at DNMT2 *target* sites	-0.409357568	0.001732
PLXNA2	668814	kahalide f	an antitumor depsipeptide in clinical trials with unclear mechanism	0.456046502	0.000991
PLXNA2	757087	Nitrogen mustard	cytotoxic organic compounds with the chloroethylamine functional group	-0.418103057	0.001344

PLXNA2	157035	Parthenolide	Anti-inflammatory product with unknown target	-0.412614505	0.004377
PLXNA2	697726	RH1	pro-drug to be activated by NQO1	-0.412631481	0.001576
PLXNA2	608210	Vinorelbine	Target microtubules	0.400038275	0.002477
PLXNA3	749226	Abiraterone	*target* androgen receptor	0.507829618	0.001135
PLXNA4	759224	Idelalisib	Target B cell receptor	0.420287248	0.00126
PLXNB1	45388	Dacarbazine	a cell cycle nonspecific antineoplastic agent	-0.411573739	0.001469
PLXNB1	668814	kahalide f	an antitumor depsipeptide in clinical trials with unclear mechanism	0.444567056	0.001373
PLXNB1	639186	Raltitrexed	a highly selective inhibitor of thymidylate synthase,	-0.480879286	0.00047
PLXNB2	638646	7-Hydroxystaurosporine	Phosphokinases inhibitor	-0.459425049	0.00102
PLXNB2	3053	Actinomycin D	a potent transcription inhibitor	-0.438765153	0.00064
PLXNB2	92859	Arsenic trioxide	induces cancer cells to undergo apoptosis	-0.484669823	0.000154
PLXNB2	706363	Arsenic trioxide	induces cancer cells to undergo apoptosis	-0.441425129	0.00066
PLXNB2	109229	Asparaginase	Targeted for ALL therapy	-0.501665774	8.19E-05
PLXNB2	45388	Dacarbazine	a cell cycle nonspecific antineoplastic agent	-0.484725657	0.000133
PLXNB2	256942	Epirubicin	an anthracycline drug used for chemotherapy	-0.453283564	0.000577
PLXNB2	141540	Etoposide	Target topoisomerase II	-0.418855637	0.001314
PLXNB2	374551	Fenretinide	inhibit cell growth through the induction of apoptosis	-0.406483608	0.001704
PLXNB2	109724	Ifosfamide	cross-linking DNA	-0.444813663	0.001363
PLXNB2	714597	Imexon	an inhibitor of ribonucleotide reductase	-0.491732124	0.000103
PLXNB2	683863	Irofulven	binds to DNA and protein *targets leading to tumor specific apoptotic cell death*	0.482282409	0.000193
PLXNB2	79037	Lomustine	It kills cancer cells by damaging the DNA and stops them from dividing	-0.410842644	0.001835
PLXNB2	755985	Nelarabine	an antineoplastic agent	-0.462192494	0.000382
PLXNB2	266046	Oxaliplatin	Triggering DNA damage	-0.445472843	0.001509
PLXNB2	759178	Pipamperone	a selective 5-HT2A, D1 and D4 antagonist	-0.417699863	0.001226
PLXNB2	713763	Rebimastat	selectively inhibits several MMPs (MMP 1, 2, 8, 9, and 14)	-0.402109209	0.003795

PLXNB3	757436	Cabozantinib	bioavailable tyrosine kinase inhibitor	0.409545238	0.001903
PLXNB3	764134	Dabrafenib	BRAF mutant inhibitor	0.504408981	0.000219
PLXNB3	679828	PD-98059		0.484272261	0.000156
PLXNB3	761431	Vemurafenib	BRAF mutant inhibitor	0.54047088	1.43E-05
PLXNC1	773263	Bafetinib	Dual Bcr-Abl and Src family kinases Lck and Lyn inhibitor	0.40416619	0.002007
PLXNC1	764134	Dabrafenib	BRAF mutant inhibitor	0.620978563	1.93E-06
PLXNC1	697979	Denileukin Diftitox Ontak	targets the CD25 subunit of the IL2 receptor	0.530792198	2.16E-05
PLXNC1	759850	Docetaxel	anti-mitotic chemotherapy	-0.53508952	2.57E-05
PLXNC1	354462	Hypothemycin	inhibitor of MEK	0.49486606	9.11E-05
PLXNC1	170984	Pimozide	selectively inhibits type 2 dopaminergic receptors	0.432692855	0.001699
PLXNC1	761431	Vemurafenib	BRAF mutant inhibitor	0.557947851	6.51E-06
PLXND1	761390	By-Product of CUDC-305	an HSP90 inhibitor	-0.465140549	0.000346

Table S6. Correlation between SEMA3 receptors and tumor stemness scores (RNAss and DNAss), tumor infiltration scores (immune score and stromal score), and tumor purity (estimate score) for breast cancer tested with Pearson Correlation tests. Note: r=correlation coefficient, and p=p-values for the correlation test. P< 1/ (11 genes x 5 scores) =0.018 as significance.

SEMA Receptors	RNAss_r	RNAss_p	DNAss_r	DNAss_p	Stromal _score_r	Stromal _score_p	Immune _score_r	Immune_ score_p	Estimate_ score_r	Estimate_ score_p
NRP1	-0.55275	<.0001	-0.1212	0.0007	0.72845	<.0001	0.31844	<.0001	0.55462	<.0001
NRP2	-0.43583	<.0001	0.00527	0.8833	0.73826	<.0001	0.38753	<.0001	0.60691	<.0001
PLXNA1	-0.08521	0.005	0.11368	0.0015	0.20934	<.0001	0.22107	<.0001	0.24108	<.0001
PLXNA2	-0.30236	<.0001	0.00905	0.8011	0.33571	<.0001	0.13077	<.0001	0.24486	<.0001

PLXNA3	-0.01554	0.609	0.0756	0.035	-0.11043	0.0002	-0.06047	0.0453	-0.097	0.0013
PLXNA4	-0.44341	<.0001	-0.14831	<.0001	0.48357	<.0001	0.18613	<.0001	0.3565	<.0001
PLXNB1	-0.2971	<.0001	-0.10152	0.0046	-0.08198	0.0066	-0.16709	<.0001	-0.1461	<.0001
PLXNB2	-0.23882	<.0001	-0.00438	0.9028	0.09359	0.0019	0.07465	0.0134	0.08927	0.0031
PLXNB3	0.07853	0.0097	0.06705	0.0616	-0.19847	<.0001	-0.06511	0.0311	-0.14011	<.0001
PLXNC1	-0.38064	<.0001	-0.11692	0.0011	0.70289	<.0001	0.37537	<.0001	0.58667	<.0001
PLXND1	-0.26379	<.0001	-0.08424	0.0188	0.37318	<.0001	0.28148	<.0001	0.35402	<.0001

© 2020 by the authors. Submitted for possible open access publication under the terms and conditions of the Creative Commons Attribution (CC BY) license (http://creativecommons.org/licenses/by/4.0/).
